# A handbook for *Rhythmic Relating* in autism: supporting social timing in play, learning and therapy

**DOI:** 10.3389/fpsyg.2024.1384068

**Published:** 2024-09-18

**Authors:** Stuart Daniel, Matthew Laurie, Jonathan T. Delafield-Butt

**Affiliations:** ^1^Laboratory for Innovation in Autism, University of Strathclyde, Glasgow, United Kingdom; ^2^British Association of Play Therapists, London, United Kingdom; ^3^Wooley Wood School, Sheffield, United Kingdom; ^4^Concept Training Ltd., Lancashire, United Kingdom; ^5^Strathclyde Institute of Education, University of Strathclyde, Glasgow, United Kingdom

**Keywords:** autism, Rhythmic Relating, education, therapy, early intervention (EI), movement, synchrony, coregulation

## Abstract

We present a handbook for Rhythmic Relating, an approach developed to support play, learning and therapy with young autistic children, unconventional communicators, and autistic people who have additional learning needs. Rhythmic Relating is based on the *Movement Sensing* perspective, a growing body of research that recognizes that autistic social difficulties stem from more basic sensory and motor differences. These sensorimotor differences directly affect embodied experience and social timing in communication. The Rhythmic Relating approach acknowledges that autistic/non-autistic interactive mismatch goes both ways and offers *bidirectional* support for social timing and expressive action in play. This handbook is presented in an accessible fashion, allowing the reader to develop at their own pace through three skill-levels and encouraging time out to practice. We begin with the basics of building rapport (seeing, copying, and celebrating interactional behaviors), introduce the basic foundations of sensory stability, and then move on to developing reciprocal play (using mirroring, matching, looping, and “Yes…and” techniques), and further to understanding sensory impetus (using sensory contours, accents and flows) and its potential in support of social timing. Rhythmic Relating is offered in support of each practitioner’s creative practice and personal sense of fun and humor in play. The model is offered as a foundation for interaction and learning, as a base practice in schools, for Occupational Therapists, Speech Therapists and Physiotherapists, and can also provide a basis for tailoring creative arts therapies when working with autistic clients.

## Introduction

### Overview

Autistic people^1^ often find themselves literally out of sync with non-autistic people, and vice versa. Interaction between neurotypes often involves considerable challenges with the *social timing*^2^ that active engagement in interactions typically depends on (for an overview, see Wimpory, 2015). Non-autistic adults have difficulties picking up subtleties of gesture from autistic children (Keen et al., 2005; Faso et al., 2015; Sheppard et al., 2016; Casartelli et al., 2020b). While autistic children have difficulties picking up subtleties of interaction, gesture, and flow from typically developing children (Rochat et al., 2013; Di Cesare et al., 2017). The difficulties go both ways.^3^ And the result: it is difficult for us to play, communicate, and learn together (Trevarthen and Delafield-Butt, 2013; Cook, 2016; Delafield-Butt et al., 2019). *Rhythmic Relating* is designed to help autistic and non-autistic people feel more in sync together, so we can share meaningful experiences, play together, and support each other in therapeutic contexts toward wellbeing^4^ (see Table 1). Given average population demographics, most carers and practitioners will be non-autistic and our model and assumptions in this paper reflect this fact. If you are an autistic carer or practitioner, Rhythmic Relating will also support your playful interactions with autistic play partners—you will simply have the advantage of autistic sensitivities and insight. Rhythmic Relating is offered in support of each practitioner’s creative practice and personal sense of fun and humor in play. The model is offered as a foundation for interaction and learning, as a base practice in schools, for Occupational Therapists, Speech Therapists and Physiotherapists, and can also provide a basis for tailoring creative arts therapies when working with autistic clients.

**Table 1:** Rhythmic Relating: an overview.

Rhythmic Relating is designed to support play, learning and therapy with young autistic children, unconventional communicators, and autistic people who have additional learning needs. Rhythmic Relating helps us to: Engage with another person; seeing, copying, and celebrating their behaviours, developing rapport and the foundations of play. Share behaviours and develop reciprocal play. We give space for the impetus inherent in the other’s spontaneous behaviours, and piggy-back on that impetus using mirroring, matching, looping, and ‘Yes…and’ techniques. Develop aspects of our behaviour which bring clarity, sensory accessibility, and impetus in support of social timing. This will include the use of certain qualities of movement, touch, and sound; and certain priorities in pattern, rhythm, repetition and novelty.

The autism research community continues to investigate the nature of sensory and movement challenges in autism^5^ and the impact these challenges might have on social timing.^6^ Into this mix, recent studies have added an examination of the relationship between *good-enough* social timing and wider relational factors such as rapport.^7^ Although we have much to learn about the fundamentals of social interaction for autistic people, it seems clear that any approach aiming to improve social timing will benefit from finding ways to facilitate *rapport* and the *sensory organization of movement* (for an overview, see Daniel et al., 2022).

The Rhythmic Relating model described in this article combines previous work on *Rhythmic Relating* and social timing (Daniel et al., 2022) and an approach called *Rapport-Based Communication* (Laurie, 2021). Rhythmic Relating aims to give you foundational skills to support your own creativity, insights, and personal sense of fun and humor in play. Here, we are attempting a *how-to-guide* and we will try to keep the ideas and the language we use as simple as possible. On occasions, we give more detail in footnotes. You can read these if interested, or skip to stick with the flow of the article. This article will build your understanding in layers (from the basics, through skill levels 1, 2, and 3), with take-home ideas at each level. It will be helpful to take in little bits at a time, stop reading for a while and try things out in practice. Then come back to this article again when you are ready. It might be that you simply do not connect with some aspects of the model, no worries, please take and use whatever works for you. In support of your learning, the online article includes live links to video examples.^8^ You can *click on the example heading*, to activate the links.

### Social timing

To give you a sense of what we mean by *social timing*, we can start with an exercise. This exercise reproduces the Still Face experiment (Tronick et al., 1978) that proved the importance of active social responses for affective connection between babies and mothers. However, even as intelligent and knowledgeable adults, the Still Face experience still works for us, because it engages our evolutionary ancient social pathways for communication and connection (see Table 2).

**Table 2:** The Still Face exercise. An optional exercise. Please feel free to skip if you prefer, it’s a helpful exercise, but not essential to your understanding of the article. Please also note, the exercise is designed to give neurotypical people an experience of a non-typical communication pattern. If you are neurodivergent or uncomfortable with it in any way, we recommend skipping this exercise.

You will need a partner. Choose who is the ‘listener’, and who is the ‘talker’ (you can swap later). Sit face-to-face in a comfortable position. Listener, you will start off with *active listening*. By this we mean, don’t say anything but do keep interested eye contact, use your body language and feel free to make social noises (“ahh ha”, “yeah”, “oooh” etc.) to be a good active listener. Talker, think of something you want to talk about – anything (this is your moment!). And start talking with your (new) friend. Listener, after about a minute or so, you will switch to *Still Face*. You will now keep your head still, facing straight ahead and gently fix your eyes on your partner’s shoulder. Stay like that, keep your face and body still (no expressions or gestures at all) and keep completely quiet. Talker, try to keep on talking naturally for another minute or so. Listener, you choose when to stop – let your partner try to keep chatting for around a minute or so (don’t leave it much longer). You are then both free to talk and move and engage with each other as you like. Continue to talk for another few minutes, so that you can find yourself in sync with your partner again. Whatever you have felt, it is important to acknowledge your feelings and to reflect on them.

When you were in the talker role, how did it feel initially when you were receiving active social prompts, and then how did it feel when your partner went Still Face? Most people feel awkward, many simply cannot continue talking. When we interact, we are continually feeding each other non-verbal social cues in a back-and-forth flow. This flow of non-verbal information is crucial to our feelings of togetherness and ease. Without it, we feel disturbed.

In a development of the Still Face experiment, known as the Double-Television experiment (Murray and Trevarthen, 1985), a baby and her mother interacted via screens. All was going great while they interacted in a natural and playfully attuned manner. The baby was happy and relaxed. Then the experimenters switched the live action to a loop of the mother’s responses from a minute earlier. What the baby was receiving was her mother’s real loving responses, but out of sync with the baby’s own live experience. The Double-Television experiment showed that an out-of-sync baby was just as disturbed as one experiencing her mother’s Still Face. Social timing is crucial to our feelings of ease and connectedness, and to our ability to share and make meaningful sense of things.

When babies experience Still Face, in the middle of active communication from their mothers, they quickly become distressed. In those initial experiments (Tronick et al., 1978), the mothers held Still Face just for a while, and quickly repaired the flowing connection with their babies. But imagine if your experience of other human beings, from when you were young onwards, was something like that Still Face experiment. Imagine if you were unable to pick-up on the social cues and flow which most of us take for granted. Imagine if you often, or always, felt out of sync with other people. And imagine how this might have affected your sense of who you are, your relationships with other people, and your ability to learn.

In using Rhythmic Relating, we support social timing in playful relationships with our autistic play partners. We use skills specifically developed to address the sensory needs of autistic people. Many of these skills are based on the science surrounding a concept we are calling *sensory impetus* (Daniel et al., 2022). Here, early on, we introduce a flavor of this idea, because this will help you to get a sense of where the article is heading. Sensory impetus will become much more tangible for you, as you work with the details and examples throughout the article at your own pace.

### Sensory impetus

When we talk about sensory impetus, we are interested in sensory experiences that are directly experienced, before conscious reflection. These direct experiences are *information-rich*: they inherently suggest movement in a particular direction, toward a particular purpose or point in space and time; they communicate the intentions of our actions; and they compel us to move. Here are a few examples of sensory impetus (in these cases, *not* from human interaction) to give you more of a sense of what we mean:

Stu (one of the authors here) has a play therapy room up some stairs, round the corner, and down the corridor from the clinic’s front door. A few years ago, a five-year-old autistic boy came to the clinic. His mother was concerned he would not come in; he was very worried about transitions. But she did not know about the big blue and yellow floor tiles laid out chess-board style down the corridor. Out in the street, the boy used the pattern of the paving slabs as an impetus; up the stairs he counted; along the corridor he felt compelled to move from blue-to-blue square until (without really realizing it) he was outside the clinic door.

In the Oliver Sacks book and film *Awakenings*, Sacks described a patient who was stuck in a sort of physical stasis, looking, to an outside observer, like a statue. The patient never moved without manipulation. Yet when Oliver, the doctor, threw a baseball toward the patient, she immediately reached up and caught it (Sacks, 1991). The perceptual looming arc of the ball coming closer compelled an automatic and perfectly-timed response.

In Edinburgh, a group of researchers have improved the physical control of many people with cerebral palsy. How did they do this? Across various tasks involving reaching to grasp an object (a perceptual and motor control-arc in space), the researchers paired the arc of the grasp with a comparable arcing sound tone (the tone flowing up and then back down, in pitch and volume, to describe the arc of the grasping action) (Schögler et al., 2017).

The perceptual flow of the patterned floor tiles, the impetus of the looming baseball, and the compelling and information-rich sound tone are just a few examples of sensory impetus.

Throughout this article, we will be interested in how we can include such sensory experiences into our playful communication. As part of the Rhythmic Relating model, we will be using them to support the well-timed moments and flows of shared experience. We will develop the use of certain types of sound, movement, physical contact, rhythm, and patterned flows of actions which communicate timing, direction, and our intentions. In this way, we can help our play partner stay in sync and get a just-ahead-in-time sense of what we are about to do.

## The basics

### Rapport

Laurie (2021) has proposed that *rapport* is the central experience underpinning a number of systems for social support in autism, including Intensive Interaction (Nind and Hewett, 2012), Responsive Communication (Caldwell et al., 2019), Floortime (Greenspan and Wieder, 2006), Son-rise (Kaufman, 2011) etc., all of which stem from, and actively facilitate, principles of early infant-caregiver interaction. In 1990, a Harvard University study investigated the nature of high-quality rapport in human interaction (Tickle-Degnen and Rosenthal, 1990). This study suggests that rapport has three essential ingredients:

1. Mutual social attention.
2. Mutual positivity.
3. Mutual co-ordination (Tickle-Degnen and Rosenthal, 1990)

Remember that it is very likely that none of these three factors will come naturally to someone autistic. We will need to find ways to support mutual co-ordination—or *social timing*. When support is given for social timing, and play-partners begin to feel a little more in sync, then the stage is set for shared experiences of attention and positivity.

Here we introduce the three Cs. Laurie (2021) developed the three Cs as a highly accessible way of putting the above *essential ingredients of rapport* into practice. With the three Cs, you can start your journey into quality interaction even when, initially, connection is very hard to find. As we describe more about Rhythmic Relating throughout this paper, every step of the way we will build on the three Cs. And, if the three Cs is your take-home message from this article—fantastic!

Each of the three Cs relates to *offers*. Offers are the in-the-moment interests and behaviors of your play partner. Some typical offers are rocking, humming, tapping, grabbing, dancing, singing etc. The three Cs are:

“*C*” *the offer*—to *see* the in-the-moment behaviors of the person as a potential starting point.

*Copy the offer*—to join in with what the person is doing using 100% of your attention. It is important to note here, right at the start, that our conceptualization of *copying* is not limited to a direct mirroring of the person’s behaviors. We can join in, in many ways. Direct mirroring is one possibility with benefits in some circumstances, limitations in others. Here, *copying* will include possibilities of connection which deliberately bypass or go beyond these limitations: *exaggerated* or *diminished mirroring*; *rhythm matching*; and *vitality matching*.

*Celebrate the offer*—to use facial expressions, body language, and tone of voice to communicate acceptance and bring warmth.

### Starting with sensory stability

*Sensory stability* is the essential ground-zero needed for autistic people to thrive and for connection to be possible. People on the autism spectrum often experience sensory disruption and feel, see, hear, and touch the world through hyper, hypo, or otherwise disorganized senses (Leekam et al., 2007; Tomchek and Dunn, 2007; Crane et al., 2009; for overviews see Ben-Sasson et al., 2019; Kojovic et al., 2019). When acute, experience like this is often frightening (Kojovic et al., 2019) and can be traumatizing (Tseng et al., 2011; Riquelme et al., 2023; Sung et al., 2024). When this is happening, interaction is impossible. Before attempting any interaction work, we suggest that you spend time observing the person you will be working with, and talking to them (if possible) and to those that know them. What is their sensory world like, and how can you design your play space to support them? There are some simple things you can look out for and do to help (see Table 3).

**Table 3:** Practical tips for sensory stability in autistic/non-autistic interaction spaces.^a^

Sensory system	Possible threats to sensory stability	Environmental adaptations to promote sensory stability
Auditory (sound)	High or low-pitched *loud* voices or sounds.^1^ Specific noises such as metallic, machine, jolting, hissing, or banging sounds, electric hums and buzzes from electronics and lights.^2,3^ Outside noises.^4,5,6^ Hearing others talking from adjacent rooms or outside.^4,5,6^	Keep the ambient sound levels low.^4,5,6^ Keep the auditory environment as simple as possible.^2,3,4,5,6^ If the child you are working with is hyper-sensitive to sounds, might noise reduction headphones be supportive?^7,8^ Initially, keep your baseline volume levels slightly below average conversational volume.^1,6^ Never shout.^1,6^ With respect to vocal tone, consider avoiding very low* or very high pitched^2,3^ registers of voice as to create an inviting tone. Adapt, as appropriate, as you get to know the child, and for varying contexts Try supplementing your verbalizations with visual supports such as drawings, symbols, lists, schedules, or written tips/instructions overview.^9^ Unplug phone chargers and other electronics chargers if not in use.*
Visual (sight)	Bright lights,^10,11,12^ high or low contrast,^13^ specific colors,^14,15^ many LEDs,^12,13^ patterned lights,^12,13^ strobing,^12,13^ flickering,^12,13^ spinning fans,* strip lights,^12,13^ some dimmer-switch lights,* light through sun-blinds (any other flickering source),^12,13^ visual complexity (wall décor, overall room set-up).^12^	Try to keep your space tidy, minimal and structured. If possible, keep the same stuff in the same places each time you interact.* Wear simple block colors without patterns or stripes.^12^ Try out different color elements. Lay out some colored fabrics somewhere in your space… is the person you are working with drawn to a specific color?^14,15^ Keep light levels low and warm.^10,11,12^ Keep, initially, to a single light source (avoiding multiple shadows and excessive lines of contrast).^13^ Have multiple light source options for hypo children to choose and control.* Be careful with the types of LEDs you use; don’t use strip-lights, dimmers, blinds etc.^10,11,12,13^ For discrimination and clarity, use color contrasting objects, consider marking boundaries of room areas visually.* It’s helpful to have a safe-space in your therapy room, such as a tent. This should be *containing* and allow for muted, dim light.*
Haptic (touch)	Highly individualized preferences… but physical contact in general and often overly gentle touch can be disturbing.^16^ Feeling disembodied and disorientated.^16^	Take time to understand and respect each child’s preferences.* As a rule-of-thumb, do not instigate physical contact but be open to playful, supportive contact when approached. If contact is initiated, try using firmer touch rather than overly gentle.* Be immediately responsive to the child’s communicated preferences regarding touch.* Have various textures, tactile objects, playdough, *fidgets* etc., available to touch, feel and explore in support of embodiment* + opinion.^17,18^
Olfactory (smell)	Smells can be overwhelming or sources of distraction.^19,20^ Or smells can support and define a stable experience.^19,20^	Try to keep your space clean and as odor-free as possible.^19,20^ Open windows for ventilation between clients.^19,20^ Do not use room-fresheners.* Do not wear scented deodorants, perfumes, or colognes.^19,20^ Have different scents available to smell, such as stickers, markers, smell bottles.^20^
Proprioception/Vestibular (body sense, positioning and alignment)	Sensorimotor dysregulation.^21,22,23^ Feeling disembodied and disorientated.^21,22,23^	Have weighted items available, such as blankets, stuffed animals, lap pads.* Have safe options for therapeutic touch available, such as a massage ball, yoga ball, textured glove etc.* Offer options for where and how to be positioned: sofa, chair, bean bag, boxes, basket, sleeping bag, sensory sock, comfortable rug.*

^a^This table is reproduced here from Daniel et al. (2024a), with author’s permission. References: ^1^Khalfa et al. (2004), ^2^Rosenhall et al. (1999), ^3^Takahashi et al. (2016), ^4^Kwakye et al. (2011), ^5^Lepisto et al. (2006), ^6^Russo et al. (2009), ^7^Pfeiffer et al. (2019), ^8^Ikuta et al. (2016), ^9^Iacono et al. (2016), ^10^Coulter (2009), ^11^Daluwatte et al. (2015), ^12^Little (2018), ^13^Koh et al. (2010), ^14^Franklin et al. (2010), ^15^Ludlow et al. (2006), ^16^Güçlü et al. (2007), ^17^Biel (2017), ^18^Laurie (2022), ^19^Crow et al. (2020), ^20^Ashwin et al. (2014), ^21^Chu (2017), ^22^Proff et al. (2022), ^23^Van Hecke et al. (2019). Other statements (marked with an *), while also reflecting extensive clinical experience (Daniel et al., 2024a), lack the backing of a reported evidence base.

## Skill level 1

### C (see) the offer

The first aim of Rhythmic Relating is for the two of you to be able to share the space comfortably. If your play partner is uncomfortable in your presence and inclined to move away, then interaction is unlikely. Tickle-Degnen and Rosenthal (1990) found that postural mirroring is the most effective technique for establishing initial connection and rapport. So, we need to look for postures, gestures, body language and other offers that we can copy. We need to *see* the offer.

Almost anything can be an offer for interaction from your play partner. Here are a few possibilities: vocalizations (a sound, something spoken); vocal patterns (humming, singing, repetitive or patterned sounds or words); movements; movement patterns (dancing, reaching, tapping or otherwise contacting their own body/your body/objects/surfaces, other repeating movement patterns); shifts in body posture/positioning/proximity; object manipulation; patterned object-play (moving, tapping, or manipulating objects); a copying of something you are doing (or have done previously), eye contact, a break in eye contact,^9^ an expectant pause.

To see the offer means to view the things your play partner does as a gesture of communication. For example, Matt (one of the authors here) worked with the parent of an autistic child who spent a significant amount of time playing with wooden blocks while turned to face a wall in the family home. The mother thought, “my child does not want to play with me.” Matt helped her to turn the situation on its head and read the situation instead as, “my child would like to play blocks with me on his own terms.” This subtle, but significant shift in perspective allowed the mother to see the offer.

### Celebrate the offer

It’s important that we value the person we are working with; value everything they do. Implicit in Rhythmic Relating is the notion that, through their offers, our play partner is making an intentional attempt to communicate. Even if we do not understand that intention, we can act *as if* each offer is rich with this potential. In doing so, we open the door to interaction and promote positive feedback for further possible engagement. So, part of celebrating the offer, is developing our ability to stay with, be patient with, and find genuine curiosity in what our play partner is doing. Our interest has to be real.

It is also important that we communicate this interest in the moment. In part, we do this through copying the offer. And, in general, we bring warmth, kindness and positivity shown in our eye contact, body language, expressions and vocalizations. Importantly, this does not mean being inauthentic, inappropriately loud, using meaningless praise, or bringing a shallow level of forced happiness into interactions. It’s okay not to be a children’s TV presenter! It’s more about real curiosity about the person in front of you, really caring for them and being interested in what they do.

### Copy the offer 1: direct mirroring

We want to piggy-back our play on the natural behaviors of our play partner. We want to copy and flow with what they are doing spontaneously. Why is this a good idea? Four answers…

The short answer is because copying is the most direct and effective way of making an initial connection with another person (Tickle-Degnen and Rosenthal, 1990). As a technique, it is probably the most effective way of establishing quick rapport and is used either consciously or unconsciously by most care givers and therapists (Burns, 1984).

The second answer is that this simple instruction—to copy the offer directly—gives practitioners something immediate and effective to do. Practitioners new to these ideas often worry about what they should be doing. Sometimes thinking about too many options, too many nuances, can leave us feeling stuck. Perhaps this is where you are in your development? If so, direct mirroring can help you bypass all of this. You have something simple to work with right now. And when you become more confident, you can move on to assimilate some of the more nuanced ideas described later in this article.

The third answer is that copying and joining-in can be understood as the foundation of adjusting our social timing to that of our play partner. Copying offers will immediately result in a shared pace to an interaction with similarly timed actions and pauses; an initial alignment which is likely to help our play partner be comfortable with our presence.

The last, slightly longer answer is that tapping into natural behaviors is the best way to unlock more behavior, novelty and playfulness. A study (with the fabulously apt title: *Give spontaneity and self-discovery a chance in ASD*) by Torres et al. (2013) showed that when we engage with spontaneous behavior patterns (often seemingly variable, seemingly random) then these patterns can morph into more predictive and reliably intentional behaviors. Engaging with spontaneous behaviors holds much more interactive potential than if we introduce new stuff from the outside.

In general, we advise starting simply. Spend some time simply sitting in the space with your partner. Keep your own energy levels low and calm, just be with them. Allow time to acclimatize and get comfortable. When we start to copy, we focus in on *one* particular offer, *one* particular aspect of our partner’s movement, sound, or object play. Here, to start off, we try *directly mirroring* the offer, literally adopting the same posture or doing the same movement or making the same noise or picking up the same sort of object. In the example above, Matt advised the mother to sit at a sensitive distance from her child, copy his sitting posture, and have some wooden blocks of her own to hand. With *real interest* in what he was doing (celebrating her child’s actions) she played with her own blocks. After some time, the child turned to her mother to connect. This was a revelation moment for her.

It is helpful to play-down the other elements of your behavior, to allow your mirroring to stand out^10^. You can also try *exaggerating* or *diminishing* your mirroring to accentuate the expressive or emotional tone of your play-partner’s offer

 (Tortora, 2006). You might try using big, distinct movements or clear, amplified sounds—but always check, does this *exaggeration* feel appropriate in the moment? Or you might use theatrical *diminishing*, taking the mirroring inwards, small, precious and special—but again, check, is this diminishing working for your play partner?

In the moment, it will be important to try out different possibilities and learn from your partner’s response. For instance, if you mirror your partner’s offer directly, this may be perceived as simplicity, clarity, and a welcome sense of recognition. Or, it risks being perceived as a patronizing over-simplification. It very much depends on the abilities and preferences of your play partner. Always stay open to their response, and to adapting in the moment. And remember this is play, and it’s about celebrating their offer. If direct mirroring is too obvious or felt as robotic, perhaps an exaggerated or diminished mirroring might work better, or perhaps you could play with *rhythm matching* or *vitality matching* (see below)? These techniques offer a way to copy and connect with more nuance. Or, of course, this approach may simply be inappropriate for your play partner on this occasion.

## Skill level 2

### Copy the offer 2: rhythm matching

Sometimes, your play partner’s offer might have a rhythmic pulse. Swaying, rocking, tapping, humming, vocalizing, singing, hand-clenching, beat-boxing, hand-flapping, shoulder shrugs, walking, running, jumping, all might have a fairly regular pulse (Bakan, 2014; Daniel, 2019; Daniel et al., 2022). We can join in and match their rhythm. There’s nothing expert here! We’re just talking basic humming, singing, tapping, creating sounds with an object, very simple beat-box, or sensitive physical strokes, squeezing, pressing, or tapping on your partner’s body. You will probably have a sense of your partners preferences and can go with those. Or you can explore different options to see what resonates. Mixing and matching sensory modalities is okay too. We can hear, see, and feel rhythm (Grahn and Brett, 2007). You can match a vocalization pattern by gently pulsing your partner’s little finger; sing their back-and-forth swaying; tap out their walking pattern on a cardboard box; beat-box their shoulder shrugs; eye-blink to their humming; click your fingers to their hand-flaps.

When we hum or sing or tap out a rhythm, we may well *accent* a beat at regular intervals. This is intuitive and natural for many people (Grahn and Brett, 2007). The simplest form of accenting is through a relative increase in volume and/or pitch.

**Audio E.G.1 Simple Time Meters for Rhythm Matching**

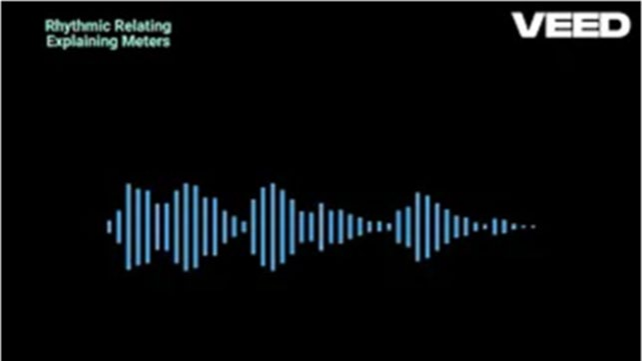
This next paragraph can be a little confusing in written form, without actually hearing the sounds we are describing; listen along with this audio example.

The intuition to accent is part of our human tendency to interpret a continuous pulse as having a rhythm with a time pattern or *meter* (Grahn and Brett, 2007). We can be aware of this and, when we rhythm match, we can choose to use a very simple,^11^ regular meter. In western music tradition, our simplest meters are: 2/4 (accented STRONG-weak-STRONG-weak; defined by a march; e.g., the Darth Vader theme in Star Wars); 3/4 (accented STRONG-weak-weak, STRONG-weak-weak; e.g., a waltz); or 4/4 (accented weak-weak- STRONG-weak, weak-weak- STRONG-weak; e.g., the straight “puh-te-Kuh-te, puh-te-Kuh-te” which opens Michael Jackson’s *Billie Jean*).

When we rhythm match, we can use our accents to add emphasis and vibrancy to a specific moment, action, or sound at regular intervals. This can bring regularity, flow and moments of shared focus into our shared rhythmic experience. Here are a few examples using rhythm matching with accents in play:

As our partner walks the edges of the room, we tap out a 2/4 march on the seat of a wooden chair. We accent every second beat with a louder tap.

**Video E.G.1. Example of Rhythm Matching in Play**

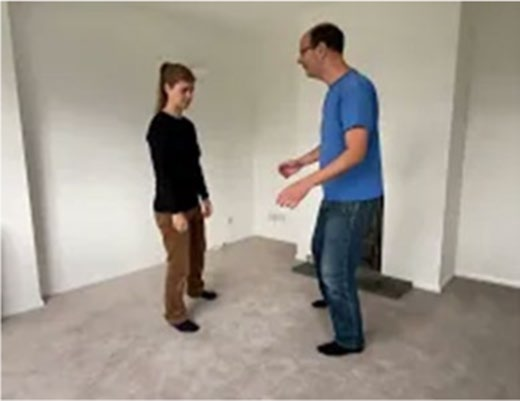
Our partner is swaying side to side in a simple swing motion. We sway along and vocalize with “tuh” sounds to a simple swinging 3/4 meter, accenting the first “tuh” of each bar by giving it more volume and force (TUH, tuh, tuh; TUH, tuh, tuh; TUH, tuh, tuh; TUH, tuh, tuh… matches left, 2, 3; right, 2, 3; left, 2, 3; right, 2, 3…).

Our partner is humming a relatively repetitious phrase. We gently pulse their little finger, pressing it gently in a slow 4/4 rhythm. We gently accent the 1 (in, 1 and 2 and 1 and…) by squeezing with a little more pressure.

**Video E.G.2. Example of Rhythm Matching in Play**

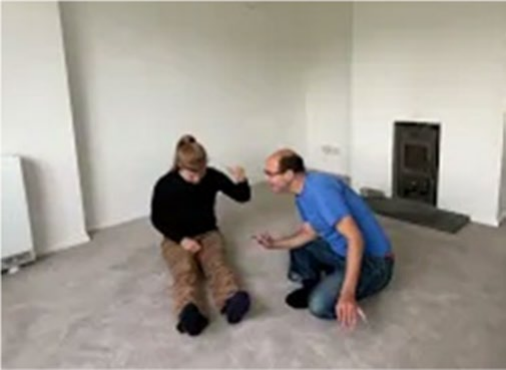
As our partner hand-flaps, we use a super-simple beat-box in a 4/4 rhythm alongside (“PUH – te – kuh – te – PUH – te – kuh – te…” matches 1 and 2 and 1 and 2 and…). We accent the 1 by giving our “PUH” a little more volume and force.

### A quick note on autism and sound

Timing delays in response to auditory information have been observed in autistic individuals across various studies (Lepisto et al., 2005, 2006; Oram Cardy et al., 2005; Russo et al., 2009; Kwakye et al., 2011; Torres et al., 2023). In the context of speech discrimination studies, Russo et al. (2009) found that the sensory experience of autistic children *without* background noise closely resembles that of typically developing children *with* background noise. Most autistic people find loudness intolerable (levels exceeding 80 dB, equivalent to shouting) (Khalfa et al., 2004), and some exhibit hypersensitivity to particular high-pitched sounds at volumes within the normal-to-mid-range (Rosenhall et al., 1999; Takahashi et al., 2014, 2016).

In support, we can use clarity and simplicity in our speech and choice of words; be patient and give time for response; avoid monotone and use a melodic “story-teller voice” or consider singing aspects of our communication; play with using rhythm matching (with simple meters and well-spaced timing); add further sensory impetus (see below); and maintain overall low-volume levels (we always suggest starting quietly, tailoring our ground-zero-volume to our partner’s needs, often below average conversational volume, i.e., <60 dB).

There’s something powerful in connecting with the rhythmic elements of autistic behavior and, also, in the possibility of adding rhythmic and melodic aspects to our communication. Rhythmic regularity helps people to predict what’s coming just-ahead-in-time^12^, and this holds true for autistic people (Knight et al., 2020). Knight et al. (ibid.) demonstrated that autistic individuals (6–21 years old), displayed no difference with encoding temporal patterns and predicting upcoming auditory events (in comparison with typically developing peers) with sounds presented in the context of simple or complex rhythms. The rhythmic context (ibid.) also nullified the delay effects demonstrated in relation to perceiving isolated sounds shown in previous studies, including, Lepisto et al., 2005, 2006; Oram Cardy et al., 2005; Russo et al., 2009; Kwakye et al., 2011; Torres et al., 2023.

### Copy the offer 3: vitality matching

We copy the offer in order to connect with our play partner and to try to communicate that we care about their choices and interests. Yet we risk over-simplification, patronizing communication, or bringing a kind of dryness to our approach and losing our playfulness. Another option, is to try matching our play partner’s *vitality* rather than attempting a direct mirror.

*Vitality* is the energy, feel, style, flavor of any particular movement (Stern, 1999, 2010). It is the *how* in the way we do something. It communicates intention, feeling, force, and the direction of our actions. For instance, you could wave your hand vigorously with excitement in greeting a friend; or slowly with tentative sadness when your friend leaves; or in tick-tock staccato to a house music beat; or in chaotic desperation in fear and panic. In these examples, the action of hand-waving is basically the same, it’s the vitality you have changed.

We experience vitality with our whole being. A vocalized “wuuuUUUPP” can compel us to stand; make an energetic star-jump and you may well feel to vocalize with a “Huh!”; on seeing a dancer slide gracefully across the floor you may well match that slide with a vocalized “aahhhh.” Vitality can be experienced, communicated, and perceived via *any* sensory modality or combination (Malloch and Trevarthen, 2009).

We can copy an offer by matching and expressing the vitality communicated by our play partner.^13^ We can match our partner’s vitality in any modality (or combination of modalities) that feels right in the moment and appropriate for our partner—we are not limited to a direct mirror. This is brilliant for two reasons: 1. We can connect without risking patronizing over-simplification; 2. We can join in with the energy and intensity of our partner’s behavior without overinvesting in any particular emotional tone (allowing us authentic connection without adding fuel to negative emotions). As with direct mirroring, we can also play with exaggerating or diminishing our vitality matching. Here are a few examples of vitality matching (from Daniel et al., 2022).

#### Within the same sensory modality…

**Video E.G.3. Example of Vitality Matching in Play**

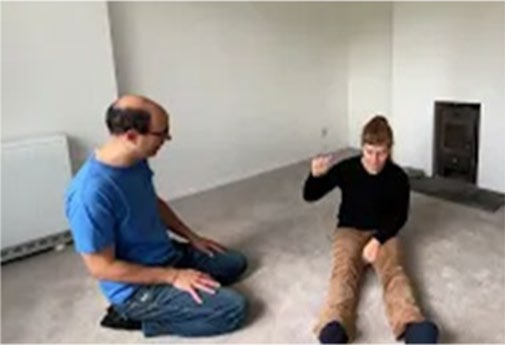
A slow rhythmic hand clench-and-release matched by a whole-body contract-then-open.

Violent jumping-stomps matched by star jumps (matching the energy release without turning up the anger).

**Video E.G.4. Example of Vitality Matching in Play**

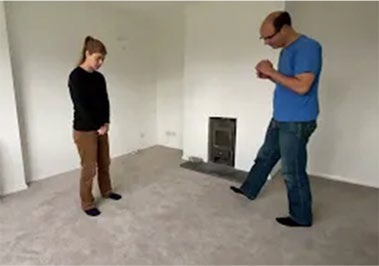
A rising anxious moan matched by bubbling sounds in a contour rising in pitch and volume (matching the rising energy but without fueling the anxiety).

#### Across different sensory modalities…

The arc of a repetitive arm movement matched by “WWWOOOhhhhhh” vocalized contours, falling in pitch and volume.

**Video E.G.5. Example of Vitality Matching in Play**

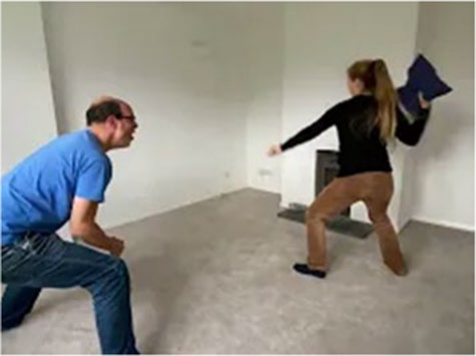
A pillow is thrown energetically at the wall, the arm movement matched in build-up by a playful vocalization (an “oooOOOOER” contour rising in pitch and volume) and then accompanied in the throw by a matching “whoooosh”.

A withdrawing sad vocalization matched with a sensitive whole-body folding over and inwards.

#### Multimodal expressions…

A toy car is energetically pushed back and forth, matched by swaying vocalizations (rising then falling contours of sound) and accompanied by left-to-right body swaying from our sitting position.

**Video E.G.6. Example of Vitality Matching in Play**

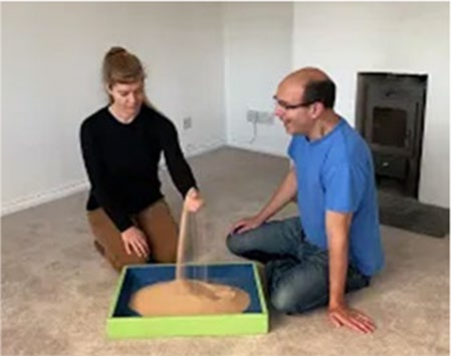
Our partner pours sand from their hand into a sand-tray, we match by a “Tssssssssshhhhhh” vocal contour (falling in volume) while we stroke our hand down our partner’s arm.

As our partner hums repetitively, we rock our upper body back-and-forth in sync, while drumming on the floor in time to the repeating pattern.

### When a game develops 1: 100% client-led

When we are patient and practicing deeply with the three Cs, we often find that two-way interactions simply emerge from the shared communicative space without us needing to contrive our actions. We trust the three Cs and do what we feel is natural. We copy our partner’s offers, wait, and celebrate. Often, at some point our partner will engage with either a slight change in their behavior or a new offer. We copy that new offer and what emerges can become a client-led back and forth interaction. It’s the beginnings of reciprocity, turn-taking and creativity in play. Here are a few examples:

**Video E.G.7. Developing a Game through Mirroring**

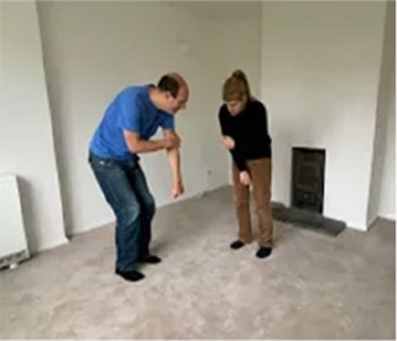
If we mirror our partner’s crouched posture for example, perhaps they feel comforted and notice our presence. When they shift a little, we copy, and perhaps they feel a sense of agency in this cause-and-effect. They crouch a little further, we copy, and this develops into a game of who-can-get-the-smallest, as we both roll-up on the floor next to each other.

If our partner is stepping back and forth across a line in the floor boards and we copy this movement for some time, perhaps they pause, then we pause too, then they start again, but this time with a little smile. We do this many times, feeling more and more in sync.

**Video E.G.8. Developing a Game through Vitality Matching**

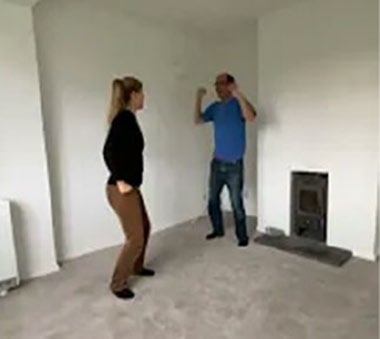
If our partner is vocalizing a loud repetitive, staccato shout (“bah, bah, bah, bah”), and we match this vitality by stretching our arms and fingers out and up in bursts of energy which correspond with each vocalisation, perhaps our partner starts to copy our actions, adding this behaviour to their vocalisations. After a while, out of tiredness or just randomly, they stop moving one arm, but continue with the other. We copy, and this develops into a reciprocal game in which our partner swaps arms and we copy in turn.

If two-way interactions are not emerging from the three Cs, we suggest thinking about the following. Firstly, consider the sensory environment and check if anything can be done to help (see *Starting with Sensory Stability*, above). Next, consider your physical proximity to your partner. Perhaps you are too far away for connection, or too close for comfort? Or perhaps your body and/or eye-line is uncomfortably above your partner? If so, adjust to match their eye-line. Or perhaps your body language is too complex, or physically or emotionally unavailable? If so, minimize, simplify, listen deeply, and be curious about your play partner. Or, as often is the case, perhaps you are failing to leave space for your partner to respond, going too fast? If we are a little impatient, we may well find ourselves out of sync with the other person and may well have missed an offer. Often, if we wait a little longer without adding anything, we find a response emerges. Being truly patient is probably the most powerful and challenging thing we need to learn.

### When a game develops 2: “Yes and…”

Another way to conceive of how to support flowing interaction is through the process, “Yes and…,” a key activity in improvisational theater (Salinsky, 2017). Here, we welcome what our partner offers (“Yes”), expand on that action or theme just a little (“and…”) and then allow space for our partner’s response. Our play partner might pick up a yellow bucket and tip some sand out of it… we could “Yes and…” this offer by finding our own bucket and tipping sand slowly… our partner may “Yes and…” this offer by catching the sand with their bucket and tipping it again… we could “Yes and…” again and catch the sand once more. When offers are responded to by actions that say, “Yes and…,” then something remarkable happens: people start to want to add to the unfolding story, to build a story together, and it feels good!

In almost all contexts, it is *crucially important* that our “Yes and…” represents just a *small* var*iation* on the theme of our partner’s offer. If our trusting relationship is very well established, big surprises and novelty can work (we do not rule this out 100%). But in almost all contexts if we over-step the mark, introduce too much novelty or too much of our own agenda, we risk shocking our partner out of connection and rapport. So, as a rule of thumb, “Yes and…” just a little.

But if our “Yes and…” does miss the mark, if our partner disconnects or if the flow becomes stilted or stuck, then we simply go back to listening carefully and trying to see the next offer. In this way, we repair the rapport. Tronick and Cohn (1989) found that typically developing infants regularly experience interactive miscoordination, yet mismatch is typically repaired close-on-instantaneously. “This constant oscillation between momentary miscoordination and interactive repair marks the essence of human dialogue, to which infants are sensitized in their earliest interactions” (Feldman, 2007b, p. 341). It can be very helpful to know that the flow of mismatch-and-repair is normal, and is itself very supportive of your developing relationship (Tronick and Cohn, 1989; Feldman, 2007a,b; Hughes, 2011).

Sometimes, as in the yellow bucket example above, your partner’s offer might be an action involving an object. But more often it will be an aspect of movement, expression, positioning, sound or rhythm. Here, it might feel less obvious how to “Yes and…” without changing things too much or adding something entirely new. We can find our way forwards by modifying our copying (our mirrored movement or sound or rhythm) just a little; *one quality* at a time. We see the offer, copy the offer, wait for response, and then copy the new offer but with a slight twist in the style, or the energy involved, the mood, the positioning, the physicality, the volume, the tempo etc. This is a big topic! Here, we offer a *few* pointers in some directions you might want to explore (see Table 4), followed by an example in play.

**Video E.G.9. Using ‘Yes…and’ in a Developing Game**

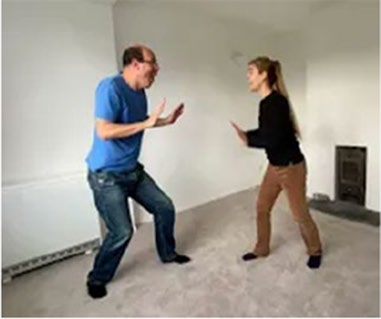
Our play partner is rocking back and forth, from back foot to front foot. A little distance away, standing in front of and facing our partner, we mirror this movement exactly. Our partner seems comfortable with this. Continuing to copy, we move a little closer. Both partner’s forward rocking movements now coincide in time. Our shared experience is of a looming forward, a well-timed meeting together, then a falling apart again. Our partner makes little giggling noises. We ‘Yes…and’ this offer, adding excited, tongue-wobbling, bubbly noises. Our sounds rise in pitch and volume to match our motion towards each other, pausing as we meet. Our partner ‘Yes…and’s our offer, vocalizing a high-pitched “hah” sound as we meet. We ‘Yes…and’ by copying the sound in sync. Our partner ‘Yes…and’s by raising their arms with the impetus of the movement and “hah” sounds. We ‘Yes…and’ by raising ours a little higher, a little closer to theirs. They raise theirs a little higher. We do so again, in turn. Our partner ‘Yes…and’s by making eye-contact, for the first time, as we meet in the up-swing of our movement. We ‘Yes…and’ by smiling and very tentatively touching their palms with our outstretched index fingers at the high point of our meeting. Our partner ‘Yes…and’s by smiling and reaching their palms forward. We ‘Yes…and’ by switching our tentative finger-reach to a tentative high-five. Our partner ‘Yes…and’s, responding with a full-on, energized high-five and shouting “HAH!”. We flow like this, with shared impetus, for a while... (to be continued…, see *When a Game Develops 5: The Story of a Game*)!

**Table 4:** Some qualities to explore and modify in “Yes…and” physical and sound play.^a^

Qualities to modify and explore	Explanation and examples
Movement (*effort*)	Modify the qualities of weight, fluidity/tension, and time in any action. E.g., you might use heavier footsteps when copying your partners walk; you might slide lightly across the floor when copying your partner’s stagnant shuffles; while rocking along with your partner you might slow down or speed up your pace.
Movement (*mood*)	Modify the emotional/energetic tone. E.g., take your walking small, gentle and whispery; take your run light, darting, magical; take your hand-clench slow, thoughtful, sacred.
Movement (*structure*)	Modify how and where your movements come from. E.g., using your body as a whole vs. in parts; considering the place of initiation of movement; playing with alternative placement or movement of your limbs in relation to your torso; exploring your upper-lower body relationship, your left to right body relationship, and your contralateral body relationship.
Movement (*space*)	Modify the ways you use proximity to your partner, sensing the effects of near, mid, far reach, and height-level changes.
Sound	In sound, you can play with small changes in *volume*; *pitch*; *timbre* (breathy, soft, or vibrato voice or sound); *tempo*; or *articulation* in the length of sounds (short and choppy, or extended and soft-edged).

^a^Regarding the *movement* qualities discussed in this table, *Rhythmic Relating* draws on ideas from *Laban/Bartenieff Movement Analysis* (LBMA) (Laban, 1976; Bartenieff and Lewis, 1980); Tortora’s related *Ways of Seeing* model (Tortora, 2006); and Eberhard-Kaechele’s developmental mirroring taxonomy (Eberhard-Kaechele, 2012). We have minimized and simplified related categories to fit the *Rhythmic Relating* priorities, and to support practitioners not trained in specific movement approaches. Regarding the *sound* qualities, we have referred to Bruscia (1987), Wigram (2004), and Geretsegger et al. (2015).

### When a game develops 3: the common third

If things feel stuck, we can wait and be with that uncertainty. When we keep sensitively with the three Cs, staying open and giving more time, our partner usually finds a way. Play usually flows when we are led by our partners offers as to the nature of our “Yes…and.” Sometimes though, if we are experimenting when things feel stuck, we can try initiating a *common third*: “the common third is about creating a commonly shared situation that… brings the two of us together. It allows us to share an activity in a way that we can both be equal, two people connected by something we both enjoy doing.”^14^ Crucially, any common third needs to be communicated as an *offer* rather than as a demand. Our partner has to be genuinely free to choose how to respond to it, or indeed free to not respond at all.

There are two possibilities here. You could offer something that you know your play partner likes, something from their behavioral repertoire, something that you have seen them like or do before. This may be a movement, sound, object, game or activity, as long as it is something you know your partner likes. Or you could offer something neutral, a bridging gesture which hopes to extend an opportunity for connection. A simple offer of a common third may be to place our open hand halfway between ourself and our play partner. We leave no sense that our partner has to do anything with it, all the while extending an opportunity for connection that was not previously present. Keeping simple, neutral and low-arousal are good rules-of-thumb here. This gesture may be received as a demand, if the hand is extended too far into our partner’s personal space, if we touch our partner without consent, or if we say something like, “it’s your turn.”^15^ We know if our partner has received the gesture as a demand if they turn away, while an offer is much more likely to engender a turning-toward. And, as always, we need to be responsive to the need to repair our connection by returning to the three “C’s” if we miss the mark.

### When a game develops 4: loops and pregnant pauses

A loop is a section of behavior or play which repeats itself, more or less, over time. Autistic people sometimes display looping behaviors: specific repetitive movements and/or sounds often referred to as “stimming” (Bakan, 2014; Kapp et al., 2019; Charlton et al., 2021; McCarty and Brumback, 2021); ritualized play patterns with hyper-focus on a particular sensation, sound, pattern or object; certain configurations of object or relational play (Daniel, 2019; Daniel et al., 2022).

As practitioners and carers, our response to loops will depend on the felt experience for our play partner in any one moment, including observable levels of dysregulation. A good example of the sensitivity needed here is in regards to stimming. *Stimming* is a commonly used term for “stereotypies”: semi-voluntary, stereotyped repetitive movements. Throughout the autistic community, stimming is widely reported as self-regulatory, self-calming and enjoyable with the only real stressful downside occurring if non-autistic people try to suppress, prevent or ridicule the actions (Bakan, 2014; Kapp et al., 2019; Charlton et al., 2021; McCarty and Brumback, 2021). In general, we should enable and support stimming. However, autistic adults report how stimming can, on occasion, become out of self-control and lead to intensity and dysregulation (Kapp et al., 2019; Charlton et al., 2021; McCarty and Brumback, 2021). We need to be sensitive to this possibility with all looping behaviors. If our partner is clearly dysregulated, if the intensity is cause for concern, or if they have expressed a prior wish for intervention, then we suggest direct (not directive) engagement with loops. Sometimes, when an autistic person is in a loop, they want to, but cannot stop. They may want your help in facilitating body-safety and control. In which case you can *offer* a behavior (remember that an *offer* is an action which the child can *choose* to engage with, or bypass easily otherwise). Here, our offer will have the potential to catalyze a change in the looping pattern, to unravel it or develop it in a different direction. Our play partner’s body and sensorimotor patterning knows what is best, yet might be stuck… For instance, if our partner is engaged in an intense pacing up-and-down the room we could try standing in front of them, or walking in front but slowing or changing the rhythm or style of our walk (this can shake-up the motor pattern), or standing or sitting close-by and engaging in something we know might grab our partner’s attention; or placing an object in the path of the looping behaviour (something which we know is of interest to our partner). If the loop changes, we can follow and flow into interaction, if there’s simply too much intensity, we can model self-calming behaviour, slowing down alongside our partner, breathing deeply, whole-body sighs, collapse gently to the floor if this feels right.

For the most part, when loops are positive or neutral experiences for our partner, we can engage in loops as wonderful way to connect and develop games. Loops have a type of rhythm (Malloch and Trevarthen, 2009; Bakan, 2014; Daniel, 2019; Daniel et al., 2022). Repetitive movements and/or sounds have their own regular beat-pattern. Looping behavioral patterns or object play have discrete behavioral steps which repeat in pattern. Our spontaneous response to feeling a pattern or beat, “is often to move at the time when the next beat is predicted” (Grahn and Brett, 2007, p. 902).^16^ In this way, loops have a kind of impetus and flow which helps us know what comes next, organize our actions just-ahead-in-time, and *compel* us into our next action.

Each loop is self-completing. By this we mean that each beat or behavioral step in the loop is *essential* to the feeling of rhythm, the impetus and flow, and the overall sense of completeness. If *our* actions become part of the pattern, *replace a beat or behavioral step*, then we can become essential *in* that rhythm and *for* that sense of completeness; a necessary *link* in our partner’s loop. From there, we can sensitively encourage the loop toward interaction and away from solo play. Working within loops in this way represents an autism-specific and powerful interpretation of the common third principle in action. Here are a few examples taken from Daniel et al., 2022:

**Video E.G.10. Becoming Part of a Loop**

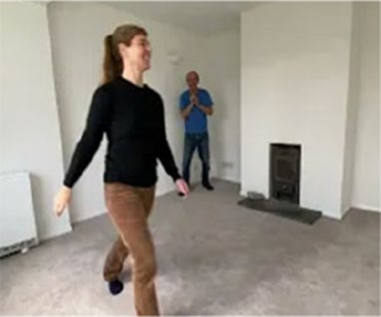
Our play partner is walking a regular circuit of the room, touching various points on the wall in a loop. We position ourself with our hand directly over one of these touch-points. When our partner next arrives at that point, perhaps they touch our hand, perhaps smile, make eye contact, do something new.

Our partner is sitting and rocking back and forth. On every fourth rock forward, we offer both our hands out, close enough to be held, not too close as to be a demand. Perhaps, after sometime, our partner reaches out and we move into rocking together hand in hand. From here we can play with all kinds of qualities of shared movement and sound.

**Video E.G.11. Becoming Part of a Loop**

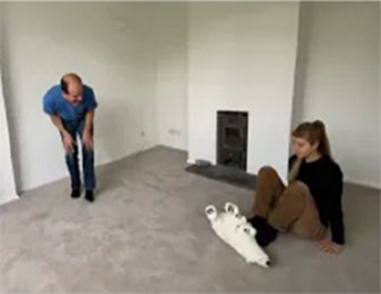
Our partner is sliding across the floor on their bottom, pushing a toy bear forward with their feet in a looping pattern (shuffle bottom forward, push bear, shuffle bottom forward…). As our partner gets ready to shuffle forward, we position ourself in their path, creating a human bridge (arched on all fours). Perhaps our partner will pass under the bridge, perhaps go round, perhaps laugh and try push us over! From here, play can develop.

Our partner is pushing a toy train around a track in a loop. Each time the train passes the station, we start to run a toy car alongside (just behind) the train. Perhaps this becomes a race, or a crash, or our partner takes the car on as a passenger.

When our movements or sounds have become integral to our partner’s loop, occasionally and sensitively we can start to play with *pregnant pauses*. Sometimes we can deliberately interject a pregnant pause into the rhythmic flow of our partner’s behavior. A pregnant pause holds a silence longer than the natural on-beat demands. It has the energy of needing to be filled. A beat or behavioral step is expected, and movement is compelled.

Pregnant pauses are an unexpected novelty for our partner. As such, we only recommend experimenting with them when your rapport is strong and you share familiar loops or behavioral patterns. We can use a pregnant pause to replace an accent in rhythm or movement, or to replace any sound or movement which has become a link in our partner’s loop. When we leave a pregnant pause, with all the rhythmic build-up of impetus, flow and expectation, something is going to happen! And often it is fun and interactive and full of potential for us to build on together.

It is surprising how long we can hold a pregnant pause for, when waiting for our partner’s response. We can play with the held duration of a pregnant pause over a range up to around 6 s. If we push the duration much past this range, we lose the rhythmical impetus of the “here and now.” “The current consensus in music psychology and cognitive neuroscience is that the ability to associate beats, or perform them meaningfully as a pulse, stops at around 6 s or 0.16 Hz… It is at this point that the mind and body can no longer “lock on”—either actively through playing, or passively through listening—to the rhythm as a pulse” (Osborne, 2017, p. 18–19).^17^

## Skill level 3

Here, the concepts we introduce begin to get a little more complex. If you feel you have enough to be getting on with, feel free to take a pause and spend some time trying out what you have already read about in practice. You can come back here any time.

We will now go on to talk in more depth about social timing, about the way neurotypical and autistic people organize their intentional movements, and then develop the use of *activation contours* and *accents*. These tools will help bring an extra level of sensory impetus and shared timing into our interactions.

### Good-enough social timing

To be very clear here, when we talk of *social timing* we are not aiming for spot-on alignment or consistent synchrony in our shared experiences. In play with typically developing infants, partners flow in and out of levels of synchrony (Feldman, 2007b).^18^ This is natural and developmentally important (Feldman, 2007b) Sometimes partners do fall directly in sync (such as the co-occurrence of social gaze, vocalizing together, the matching of arousal level, or the coordination of parent affectionate touch with infant social gaze). More often partners experience a kind of c*omplementarity*; playing and teasing around a game within a shared time-frame (Feldman, 2007b). And indeed, partners are often out of sync, leading naturally to moments of interactive repair which constitute important practice steps in the development of robust social skills (Tronick and Cohn, 1989; Feldman, 2007a,b). We are looking for *good-enough social timing*.

We need to share *moments* where our intentional actions meet. Sharing our intentions, through timing and sequencing our actions together, is crucial for the development of togetherness and meaning (Fantasia and Delafield-Butt, 2023). For example, if your partner is reaching to touch a ball, then truly connecting means to share that intention, to move together toward it, to touch the ball together in space and time. Or if you are playfully high-fiving, then connecting means you share the intention and organized movement patterns which bring your hands together. We need also to share *flows* over time in which our intentional actions are complimentary, run alongside, connect with and develop each other. For example, if you are rocking together, then connecting means that both of you share the intention, the rhythm, and the movement flow which enables your bodies to be coupled in that movement. If your partner is walking slowly, then connecting means to share the arc of your steps together; arcs which culminate in your steps happening together.

### The way we organize movements and social timing

Neurotypical individuals organize their intentional movements just-ahead-in-time. A motor image is generated in the brain just before action. It’s a neural image full of predictive information. In reaching out an arm to grasp an object, for example, the motor image pre-empts the movement needed and builds in an integrated sense of specific intention (i.e., to reach the object in a way advantageous of the type of object and the intended relationship to the object—is it heavy, delicate, difficult to hold, full of water; is it to be held gently, turned upside down, squashed?).

Often, these motor images are *contours*; contours of energy, direction, force, and intention (Lee, 1998; Stern, 1999; Rousanoglou and Boudolos, 2006; Delafield-Butt et al., 2018). In our reach-to-grasp example, the motor image is a contour of energetic enervation: rising on initiation, increasing to reach, falling in expectation to control the timing and nature of contact with the object. The contour gives the organization and sensory impetus for the action; an action with an intentional goal in space and time. When actually reaching for that object in real time, neurotypical individuals activate the motor image, start to move, and then use continuous sensory feedback to adjust and assess the image in action. It’s through combining our motor images (movement contours) with our real-time sense of an object in motion (perceptual contours), that we are able to sync-up with that object when, for instance, catching a ball (Lee, 1998). And, most importantly for us here, it’s this coupling of movement and perceptual contours which enables neurotypical individuals to couple their moving actions with those of another person (Lee, 1998; Rousanoglou and Boudolos, 2006; Delafield-Butt et al., 2018). It’s when our intentional actions are aligned in this way that we are able to *feel* and *share* the intentions inherent in another person’s actions. Good-enough social timing and our shared sense of play relies on this happening over and over in real-time.

Importantly, autistic individuals have challenges in integrating information for the generation and implementation of motor images for intentional actions^19^ (Mari et al., 2003; Rinehart et al., 2006; Cattaneo et al., 2007; Fabbri-Destro et al., 2009; Chua et al., 2022; Fourie et al., 2024; for an overview, see Daniel et al., 2022). In play with autistic partners, we need tools to magnify the clarity of *our* actions, the clarity of our own intentional movements. We also need tools to help our autistic partners organize, enact and compel *their own* intentional motor contours. And we need tools to bring our movement contours into alignment for good-enough social timing.

### Activation contours

In the introduction, we mentioned an Edinburgh study in which sound tones were used to help people with cerebral palsy control their reach-to-grasp arm movements. How did this work? The sound used was a tonal flow, a “wooooOOOOOooooo” type sound, flowing up and then back down to stillness. This tonal flow was designed to imitate the neural flow of energy used by animals of all kinds when generating action (Delafield-Butt et al., 2010, 2012), including human babies (Delafield-Butt et al., 2018) and in creating music together (Schögler et al., 2008). When the tone was paired with the participant’s real-time attempts at reach-to-grasp., the tonal flow gave impetus and organization to the participant’s motor image (their motor contour). The results showed greatly improved movement control and synchrony (Schögler et al., 2017). The type of sound tone used in these studies is an example of an *activation contour*.

Activation contours contain, “…the felt experience of force… with a temporal contour and a sense of aliveness, of going somewhere” (Stern, 2010, p. 3).^20^ Importantly for us, in “going somewhere,” they point toward, and compel us toward a point in space and time and a focus for intention. They contain a sense of what’s coming next. Activation contours are often *bell-shaped* curves (like the sound tone described above, or the motor contour when we reach-to-grasp an object). They can also be *upwards* contours (starting low in energy and rising to a point in space, time, and intensity) or *downwards* (starting with higher energy and falling or fading to a point in space, time, and intensity). Activation contours can be expressed in *any sensory modality* or combination of modalities. Here are a few playful examples of the use of activation contours to facilitate shared moments, flows, and intentions:

If our partner is reaching to touch a ball, we can playfully exaggerate our own movement arc toward the ball and add a “wwoooOOOOOoooo” sound effect (a bell-shaped acoustic activation contour), all in real-time sync with their intentional movement. Our movement and sound contours *match* and *guide* our partners movement contour, with the climax of all contours landing exactly on contact with the ball.

**Video E.G.12. Using Vocal Activation Contours**

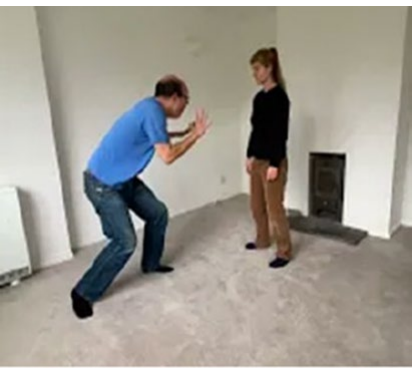
If we are playfully high-fiving with our partner, we can first isolate the movement pattern (pausing for a moment, literally letting our body become neutral), then start the movement from a low, contracted crouch position. In sync, we can exaggerate our movement contour up through a whole-body expansion, on to an arm extension, all augmented by a rising “oooooOOOOOHH” acoustic activation contour. Our movement and sound contours flow up to a point in space and time – the moment for the high-five. The whole sensory flow has impetus and information to guide our partner into a synchronous high-five of their own.

If our partner is walking slowly, we can match the movement contours of each step (and the overall rhythm) with theatrically diminished steps of our own. We can emphasize each movement contour, and the moment each step contacts the floor, with precise, careful, magical tip-toeing. And we can add further sensory impetus, clarity and mood through adding whispery “SSSSSSsssshhhhh” falling sound effects as acoustic activation contours guiding each step to contact with the floor.

We can also play with tactile contours—carefully, and always checking if physical contact seems appropriate in any one moment. We can generate supportive felt contours in our partner through touch or through supported movement.

**Video E.G.13. Using Tactile Activation Contours**

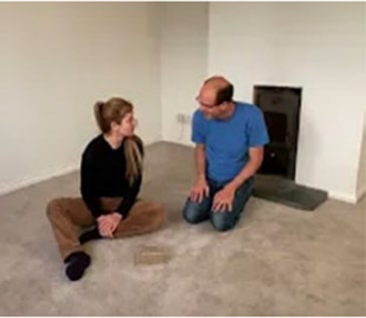
We might stroke our hand down the side of our partner’s forearm, while vocalizing an upwards “wooooOOOO” contour, to compel us to move together, in sync, to press a button, nudge a ball forward, or push a tower of blocks down.

Next, we explore the types and qualities of sound which are particularly useful when using acoustic activation contours with autistic individuals. We will also build on our concept of accents, which will be particularly relevant when we go on to develop our practice of rhythm matching a little later on.

### The neuroscience of sensory impetus in sound

Recently, a computational method for automatically categorizing music (X-System), has demonstrated success in predicting emotional, arousal, and mood responses to music (Sice et al., 2020). The X-System analyses distinct attributes of sounds that humans have evolved *to be moved* by. These sounds include the *acoustic startle response* (ASR) and *acoustic activation contours*. They elicit highly immediate and direct movement responses.

The human acoustic startle response (ASR) represents a neurophysiologically immediate reaction to sudden and unexpected auditory stimuli.^21^ Acoustic activation contours are evolutionarily significant sounds indicative of the positioning and movement of the human body in space and time (from sudden approach, to slowly moving away). These include separation cries (Panksepp, 2003), or the hissing of snakes (Erlich et al., 2013), and rapidly approaching sounds, glides, falling, fast crescendos, or bursts of sound (Osborne, 2009).^22^

Above, we mentioned various studies which have reported auditory timing delays in autism. If used sensitively, it is possible that we can use the activation and immediacy of *acoustic activation contours* and *ASR-type sounds* to bypass the neural pathways involved in these delays (see Daniel et al., 2022 for a detailed analysis). If so, we may be able to add clarity and impetus to our own movements and sounds, and augment our partner’s offers of movement and sound, in ways more immediate, more in sync, and more compelling than otherwise available.

In music, the *sensitive* use of such activating sounds involves modulation, context, timing, and expectation. With this sensitivity, music deliberately takes the “dangerous” edge off acoustic activation and startle (the fight/flight/freeze edge) and leverages that energy for *joy* (bursts, fast upwards contours, energetic building crescendos, sounds full of fun, randomized internal movement), *wonderment* (subtle downwards contours, quick fades, quiet hisses, whispers, jangles), and *anticipation* (contours in volume and pitch leading somewhere, pregnant pauses, shifts in tempo) (Osborne, 2020).

If we want to play with using activating sounds in support of social timing, we need to be conscious that these experiences are on the edge of edgy! They are neurologically reminiscent of fight/flight/freeze sounds. This is what makes them activating and exciting. But we need to be mindful of the parameters that make them fun as opposed to scary. For autistic people specifically, what takes the dangerous edge off these sounds, and makes them sounds of delight, intrigue, and anticipation is: a safe relationship; familiarity in context; familiarity of patterns of behavior and play (repetition^23^ and small-step changes); low-level ambient and spoken sound (<60 dB); and *relatively* low-level accent volumes (*relative* acoustic startle—just above 60 dB) (for further detail see, Daniel et al., 2022).

### Accents and acoustic activation contours in play

Perfect for acoustic activation contours are any sounds with lots of inherent movement: glides (“sssssssooooo”); crescendos (including upwards “whhhHHOOOOP” effects, animals “wooOOF,” growl, squark); decrescendos (including downwards expressive sighs, “ZZZEEEEeeeooooo” effects); variations on playful hissing (“ssss,” “ssshhh,” blowing sounds); sounds with high levels of randomized internal movement (bubbling noises, raspberries, tongue wobbles); contoured spoken words; contoured sound effects (cars going past go “*vrroOOOoom,”* contoured “woooOOOOooo” effects; cartoon characters are literally designed for this, Scooby-doo’s “zzoiiEEKKS! for example; and if you can manage an elephant trumpet…!).

We can explore the playful “shock” factor of *relative acoustic startle* in our accents. We can choose our tones and relative volume levels to modulate ASR-type effects down to *happy-startles*—sonic moments full of wonder, surprise and delight. Initially, just over 60 dB, we can consider using drum-like bass tones; the quick hiss-factor of a high-hat-like-tone; the surprise of a higher-pitched machine-like pulse (e.g., a laser gun sound effect); “magical” pulses like bell and triangle tings; animal noises; cartoon character refrains (Homer Simpson’s “Doh!” as a perfect beat); machine and vehicle sounds; impressions; and focus words.

### Accents and activation contours in rhythm matching

Developing our examples from, *Copy the Offer 2: Rhythm Matching*, we now include the sensitive use of accents with relative acoustic startle…

**Video E.G.14. Using Accents in Rhythm Matching**

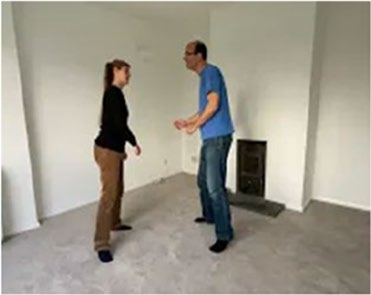
In our swaying example, we can add some sparkle to our accents. On every fourth bar, we accent the 1 with a relatively loud and bright accented “BING!” (TUH, tuh, tuh; TUH, tuh, tuh; TUH, tuh, tuh; “BING!”, tuh, tuh…). Our partner may feel connected with us due to our shared swaying. And the “Bing!” (as it is pitched at a sensitive, relatively loud volume; is well-spaced, and comes as part of the felt rhythm of the swaying pattern) may be heard directly, in-sync, and produce a feeling of pleasant intrigue.

In our example where our partner is walking, we keep tapping out the 2/4 march but replace every other beat with a relatively loud, comical “Oooh!.” We get, tap—“Oooh!”—tap—“Oooh!”—tap.

**Video E.G.15. Using Accents in Rhythm Matching**

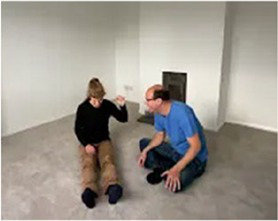
In our example where we’re using super-simple beat-box to match our partner’s hand-flaps, knowing our partner likes dogs and finds dog noises funny, we add “WOOF!” on the 1, instead of the accented Puh. We get, “WOOF! – te – kuh – te – WOOF! – te – kuh – te…”

Or we can turn the volume down low in gentleness and magical intrigue…

Our partner continues humming. We continue to gently pulse their little finger, pressing it gently in a slow 4/4 rhythm. Every fourth bar, we add extra accent to the already accented 1 by blowing on our partner’s hand (a tactile accent with a whispery sound).

We can also play with replacing a few beats or a full bar of our rhythm pattern with an activation contour. The contour could be in sound, or movement, or touch, or any combination. Such contours will guide us and our partner to land together on-beat with our accent. They compel us to move together.

**Video E.G.16. Using Activation Contours in Rhythm Matching**

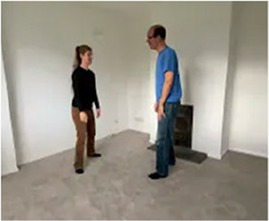
Developing the example from just above, our swaying rhythm becomes, TUH, tuh, tuh; TUH, tuh, tuh; TUH, tuh, tuh; “BING!”, tuh, tuh; TUH, tuh, tuh; TUH, tuh, tuh; “whooooOOOOO”; “BING!”, tuh, tuh. We use an upwards vocal contour to guide us to the shared accent, “BING!”. We might also exaggerate our swinging movement in the lead up to the accent, accompanying and emphasizing the sound contour with our own movement contour.

### Loops and activation contours

A powerful use of activation contours is *in combination* with our link role in our partner’s loops. We can use activation contours to compel our combined attention toward the link, and give energy and impetus to that shared moment. Developing the above examples from *When a Game Develops 4: Loops and Pregnant Pauses*, we add activation contours:

**Video E.G.17. Using Activation Contours as part of a Loop**

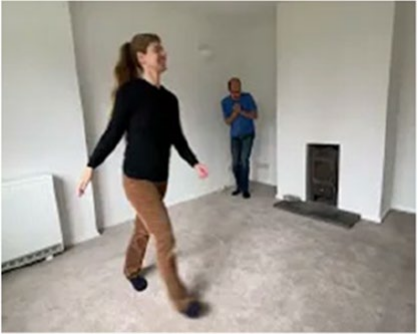
Our partner continues to walk a regular circuit of the room, touching various points on the wall in a loop. They are comfortable with, and are a little intrigued by, our hand appearing at a certain touch point in the circ uit. A couple of times they have touched our hand and smiled. This time, as our partner walks towards the shared touch point, we walk alongside them. As we do, we start low and crouched, and then grow to our full out-stretched height, culminating with a wide reach to the shared touch point. We are using a physical activation contour, an outwards-upwards contour of our body in motion; a contour that leads naturally to the shared touch point. Accompanying the physical contour we use a vocal upwards contour, “ooooOOOOOO!” slowly rising in volume and coming to rest in sync with the shared touch point and the culmination of our physical contour.

Our partner has, after some time, taken our hands and we sit facing each other and rocking back and forth in a shared rowing motion. We add upwards, then downwards vocal contours to accompany each forward and backward motion (as experienced by our partner). We use whispery, soft timbre sounds like waves as they move in and out on the shore.

**Video E.G.18. Using Activation Contours as part of a Loop**

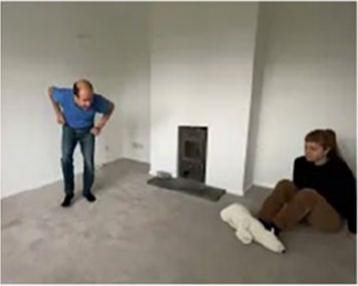
Each time we create the human bridge for our partner, they shuffle forwards through the bridge pushing the toy bear in front of them. We add a bell-curved vocal contour, curving up as our partner approaches and enters the bridge, curving down again as they leave and pass through. Our contour is a lively, wobbly, jangly sound effect made as we quickly waggle our tongue from side to side.

Our partner has developed the moment of meeting between their train and our car into a regular crash point in their looping play. To add energy and impetus, as we move the car up toward the crash point, we add the cartoon sound effect of an object falling from the sky, “SSSHHHOOoooowwww”! The lowest point of the contour is synchronous with the crash point.

### Loops, activation contours, and changing things up

Along with activation contours, we can start to play with replacing our link-moment in our partners loop, with a pregnant pause. Developing two of the above examples:

With the impetus of the car moving and the accompanying vocal contour the car crash becomes essential and expected, but this time we playfully stop just before the crash (our play partner looks surprised and engages in a brand-new way).

**Video E.G.19. Using Activation Contours and Pregnant Pauses as part of a Loop**

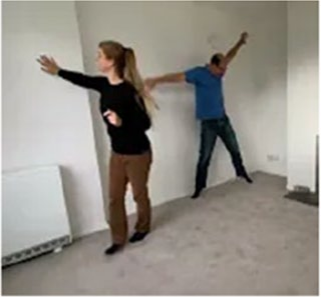
Our play partner gets used to our physical and vocal contours leading in expectation to our hands meeting at the touch point (the link) but this time we keep our hand back (compelled to act, and without the expected resolution, our partner does something new, something we can playfully build on).

Or, instead of a pregnant pause, when we have a good level of rapport and a well-established loop, we could playfully, cheekily, do something completely unexpected! Developing two of the above examples:

**Video E.G.20. Using Activation Contours and Novelty as part of a Loop**

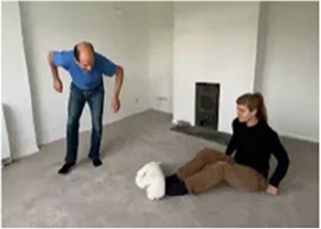
All of our sensory impetus has led to our play partner expecting, and being drawn to pass under our human bridge, but this time we shrink in size making it very difficult for them to squeeze on through (a new game develops).

Our link has brought us and our play partner together in a hand-held rocking motion, but this time we just fall over to the side in the middle of it all (our partner laughs and interacts in a new way).

All these examples come with some risk of shocking or jarring our play partner out of an easy flowing connection with us. But, done sensitively the risk can have a huge pay off with new levels of play and relationship. And please remember, if that jarring does happen, we can simply return to the three C’s and repair our connection. No worries.

### When a game develops 5: the story of a game

**Figure 1:**
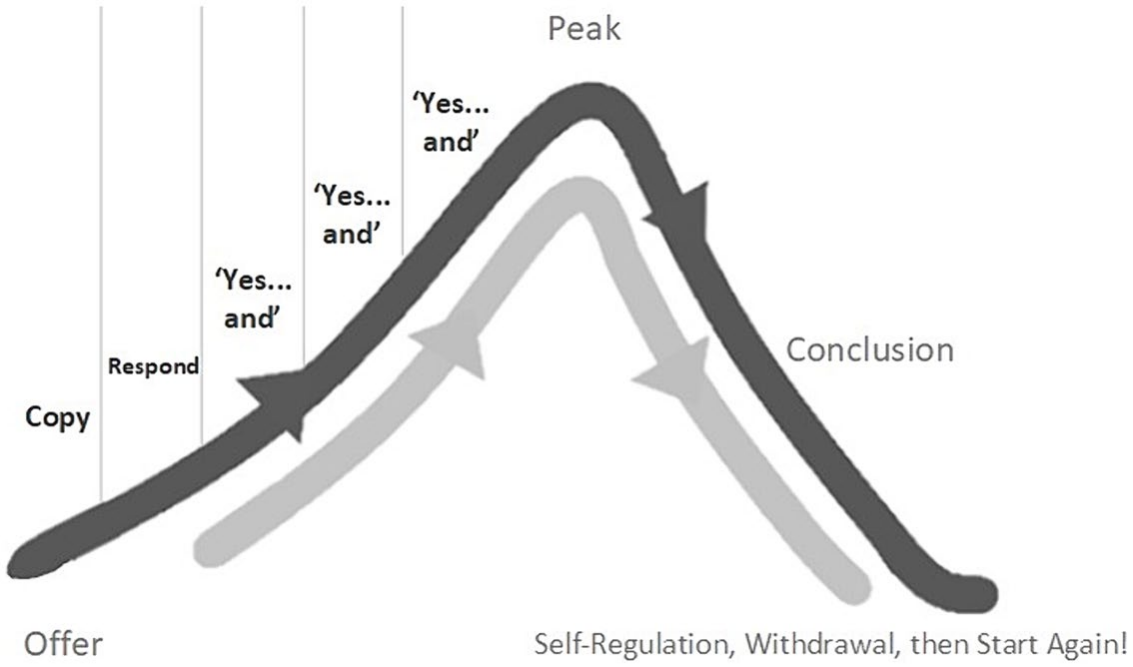
The Bell-curve of “Yes...and” play.

Stern (1974) described how, in the first 18 months or so of typical development, play between young children and their carers often takes the loose structure of a skewed bell-curve (see Figure 1).

After an initial orientation around an offer, Stern noticed that games tend to build with a phase of growing momentum, flowing up toward a “peak.” He also noted that, nested within this phase, play partners tend to share several “Yes…and” moments which provide the impetus of the game. Each “Yes…and” tends to build just a little on the previous, adding a little more intrigue, curiosity, energy, often developing the shared movement or sound just a little. This natural pattern of early game play supports social timing between play partners in several ways: impetus is added through each “Yes…and”; the expected structural flow helps partners feel and anticipate what is coming next; moods are built and shared, each with their own felt ambiance and expectations. If we play with these elements in our interactions, we will be helping our autistic partner to feel what’s about to come next, to anticipate it, to plan their actions, and to feel in sync with us.

Significantly, these “Yes…and” build-ups do not go on indefinitely. They form units or “stories” that start, build, reach a peak-moment of excitation, then draw to a natural conclusion (Stern, 1999; Malloch and Trevarthen, 2009; Delafield-Butt, 2018; McGowan and Delafield-Butt, 2022). Typically developing young children regulate these bell-curves of play by switching their behavior at the “peak.” If the game has been defined by increasing joy, perhaps the peak will be in laughter and physical release. If the game is one defined by magical curiosity and whispery wonderment, then perhaps the peak is in quiet shared eye contact or a whole-body contraction. If the game has been an exploration of anger, perhaps the peak is in a burst of physical power and release or a pushing out or through.

After the peak, typically developing young children will initiate a short period of conclusion and then withdraw from active interaction; a chill-out self-regulatory moment. But they are always ready for the next round, often literally with the words “again, again!” These moments of chill-out withdrawal are crucial practice-steps in the child’s journey toward self-regulation.

In our play with autistic play partners, it’s essential we get to know and feel the differences between these positive periods of self-regulation and moments in which our “Yes…and” offers have missed the mark. The former are usually short periods of active down-time. Your partner might turn away briefly, break eye-contact, and relax their anticipation. But throughout, there is a sense of healthy connection. Your partner will be immediately back to engage when they are ready. The latter (a moment in need of repair), will probably look more obvious and will lead to a more significant jolt in the interaction. Your partner is likely to disengage for longer, distancing themselves, the mood shifting; they might perhaps move away or engage in something entirely different.

It’s also important we understand that it’s likely our autistic play partner will find it difficult to instigate chill-out moments for themselves. We can model self-regulatory behaviors as part of our play.

**Video E.G.21. Using ‘Yes…and’, and Modelling Self-Regulation**

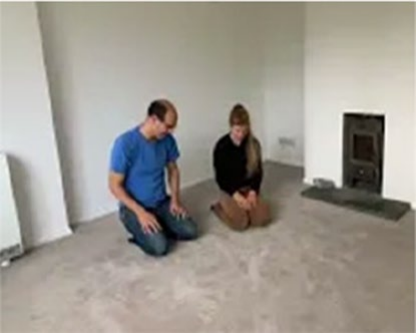
In concluding the example from, When a Game Develops 2: “Yes and…”, here we show how we can sensitively encourage and model self-regulation in play… Our partner ‘Yes…and’s by smiling and reaching their palms forward. We ‘Yes…and’ by switching our tentative finger-reach to a tentative high-five. Our partner ‘Yes…and’s, responding with a full-on, energized high-five and shouting “HAH!”. We flow like this, with shared impetus, for a while. The energy builds. To us, it feels like a ‘peak’ moment and we decide to model and support a ‘conclusion’ and on into a period of ‘withdrawal’ and regulatory chill-out (see Fig. 3). After an energetic high-five, we add a downwards acoustic activation contour (“WWWHHHHOOOooooooooo”) and slowly collapse to the floor while encouraging our partner to come with us. They do. We sit for a while and we model careful, longer cycles of breath. Then our partner stands and we start over.

## Discussion

In this article, we have presented Rhythmic Relating in support of playful interactions between autistic people and non-autistic carers or practitioners. Rhythmic Relating is based on a growing body of literature that recognizes that autistic social difficulties stem from more basic sensory and motor differences. These sensorimotor differences directly affect embodied experience and social timing in communication. The Rhythmic Relating model focuses on *bidirectional* support for the fundamentals of sensorimotor organization; addressing interactive mismatch and facilitating expressive action, social timing and play. When autistic people and their play partners are experiencing good-enough social timing—experiencing shared moments, flows and feelings—then the dynamics are right for rapport, co-regulation, shared meaning and learning.

Rhythmic Relating aims to give you skills to support your own creativity, insights, and personal sense of fun and humor in play. Please use what works for you, taking the model as a starting point and not a limiting prescription. The model is offered as a foundation for interaction and learning, as a base practice in schools, for Occupational Therapists, Speech Therapists and Physiotherapists, and can also provide a basis for tailoring creative arts therapies when working with autistic clients. To date, two pilot intervention studies have demonstrated significant positive effects on Emotion Regulation measures for young autistic children receiving a combination of Rhythmic Relating and Child-Centered Play Therapy (Daniel et al., 2023; Daniel et al., 2024b). Future work with case study designs and large-scale efficacy studies will support improved understanding of the role of timing and shared social rhythms in autism interaction, while also testing and developing the Rhythmic Relating model across a range of contexts.

## Data availability statement

The original contributions presented in the study are included in the article/supplementary material, further inquiries can be directed to the corresponding authors.

## Ethics statement

Written informed consent was obtained from the individual(s) for the publication of any potentially identifiable images or data included in this article.

## Author contributions

SD: Conceptualization, Writing – original draft, Writing – review & editing. ML: Conceptualization, Writing – original draft, Writing – review & editing. JD-B: Writing – original draft, Writing – review & editing.

## Footnotes

^1^The authors note the sensitively evolving nature of the discussion on societal, community, and individual preferences with regards to the choice between person-first and identity-first language when talking about autism. For an overview of this discussion, please see: https://www.nih.gov/about-nih/what-we-do/science-health-public-trust/perspectives/writing-respectfully-person-first-identity-first-language. We note that at all times, individual preferences should be prioritized in direct communication. When writing, it is necessary for authors to choose an option. Currently, many sources of information and opinion are focussing on a very positive community trend opposing deficit or disorder models, and drawing attention to autism as a highly positive factor of identity. For an overview of this position, please see: https://www.autism.org.uk/what-we-do/help-and-support/how-to-talk-about-autism. As such, we will be employing identity-first language throughout this article. We ask for your patience and understanding if this is not your personal preference. Thank you.

^2^*Social Timing* encompasses the processes and information-sharing involved in the meaningful organisation and production of temporally-matched movements and sounds that occur during interaction.

^3^The Double Empathy Problem (Milton et al., 2022) and the Interactional Heterogeneity Hypothesis (Georgescu et al., 2020), predict that social timing challenges in autistic/non-autistic pairings are due to *interpersonal mismatch*, as opposed to impairments isolated in the autistic partner—a prediction supported by evidence that autistic people prefer interactions with other autistic people, finding the quality of interaction considerably easier, from Sasson et al. (2017), Bolis and Schilbach (2018), Morrison et al. (2019), Crompton et al. (2020), Georgescu et al. (2020), and Wilson and Bishop (2021).

^4^We encapsulate *wellbeing* as the movement towards happiness and *good-enough self-regulation* which, in turn, we define as, the ability to attain and maintain a *good-enough* state of arousal fit for task/environment/moment. Here we are suggesting co-regulatory play is the *primary* way human children learn to self-regulate. Through physical touch, voice and early play, typically developing infants and their attachment figures engage in co-regulation. *Co-regulation* is when one person’s nervous system supports another’s nervous system through vulnerability and back to okayness. During play, typically developing infants regularly push the boundaries of safety—think of the uncertainty inherent in the games “hide-n-seek” and “peek-a-boo.” In these games, the infant and adult travel together, through vulnerability (on the edge of dysregulation) and back down to good-enough regulation. In this way, the infant’s nervous system is trained to down-regulate to healthy working levels (Porges, 2021). Co-regulatory play relies on a degree of togetherness between play partners; *good-enough social timing* is essential. We also note there are other routes to learning to self-regulate, such as, time with a calming animal, sensitive physical pressure, taught techniques (such as breath work), rhythmical physicality etc. These can also be highly beneficial for autistic individuals.

^5^Movement is fundamental to the human mind. Through movement we experience, interact, and learn. Importantly, neurotypical people generate movements *expectant* of their sensory contingencies, their sensory responses. For example, it is neurotypical to grasp a glass of water *expecting* that glass to be at the end of a 1-s-long reach, with its familiar tactile sensations and resistances at the fingertips, and weight of the glass as it is picked up to drink. This fundamental *prospective* sensorimotor organisation is impacted in autism. Evidence demonstrates that movements are organised with a different awareness of their anticipated effects in autism (for an overview, see Torres et al., 2013; Delafield-Butt and Trevarthen, 2017; + click here for a latest exploration of *The Movement Perspective* online), which challenges expectations and can lead to distress, social timing challenges, and withdrawal (for an overview, see Trevarthen and Delafield-Butt, 2013).

^6^Social Motor Synchrony (SMS) challenges have been demonstrated in various studies involving mixed pairings (autistic/non-autistic) as opposed to non-autistic pairings (Fitzpatrick et al., 2016; Kaur et al., 2018; Su et al., 2020; Zampella et al., 2020; for an overview, see Glass and Yuill, 2023; McNaughton and Redcay, 2020). The degree of SMS disruption evidenced in autistic-non-autistic interactions correlates significantly with severity of autism diagnosis (Nebel et al., 2016; Kaur et al., 2018; Su et al., 2020; for an overview, see McNaughton and Redcay, 2020).

^7^Synchrony is associated with *rapport* (Tickle-Degnen and Rosenthal, 1990; Au and Lo, 2020), and is quickly disrupted by its opposite: social anxiety (Asher et al., 2020). Many of the above reported SMS study designs (see footnote 6) did not allow for acclimatization, sensory stability, or relational rapport (for an overview, see Glass and Yuill, 2023). When these factors were facilitated via familiar settings, personalized tasks, and relationship building (*ibid.*), and in particular, via the Intensive Interaction model (Glass and Yuill, 2024), tentative findings suggest that observed synchrony differences in autistic/non-autistic dyads (as opposed to non-autistic dyads) are minimized. Also, note here, the fact that SMS study designs have consistently failed to include autistic-autistic dyads within their design—and the significance of this fact, given the strong evidence for the Interactional Heterogeneity Hypothesis (see footnote 3).

^8^All the examples featured in this paper (video and text) are generic. They are inspired by the themes and content of real play sessions, informed by clinical practice, but are non-specific and non-identifiable to any one person. Both video participants (Lea Zaccari and Stuart Daniel) have given their full permission to appear publicly (on YouTube) and via the platform of this article.

^9^A well understood aspect of autism is that eye-contact is difficult for many autistic individuals. It is very important to note that a lack of eye contact does not mean that an autistic person is not listening or paying attention.

^10^Di Cesare et al. (2017) have shown that autistic children have difficulties in perceiving vitality-form differences between two contiguous stimuli (smallest change detected at >100 ms apart). This suggests that, “during action observation, children with ASD need greater stimuli variations than TD children to detect their differences in terms of vitality forms” (Di Cesare et al., 2017, p. 8).

^11^Simple meter patterns, as opposed to complex ones, have been shown to improve synchronization dynamics (Patel et al., 2005).

^12^Here, our concept of rhythm is *information-rich* with sensory impetus and flow (including *temporal accents*—e.g., the future-oriented emphasis of an iambic pentameter (for more details please see Daniel et al., 2022), and *non-temporal intensity accents*—use of changes in volume, pitch, timbre; and *acoustic activation contours* (all explained later in Skill Level 3). Dry metronomic time keeping is not enough to support the good-enough social timing needed for interactive play. It is the use of *information-rich* rhythms, as opposed to information-dry metronomic rhythms, that makes the difference between success and failure in synchronization tasks in autism studies (*tapping studies*, Tryfon et al., 2017; Yoo and Yoon, 2019; + for analysis see Daniel et al., 2022).

^13^The authors are aware of the challenges of communicating vitality in autistic-non-autistic interactions. Autistic children, as compared with typically developing (TD) children, demonstrate significant differences in vitality-form expression (Casartelli et al., 2020a) and challenges in recognition of TD vitality-forms (*imitation studies*, Hobson and Lee, 1999; *similarity judgments*, Rochat et al., 2013; *immediate evaluations*, Di Cesare et al., 2017, 2020). Di Cesare et al. (2017) have shown that children with “high functioning” autism have difficulties in perceiving vitality-form differences between two contiguous stimuli (smallest change detected at >100 ms apart). This suggests that, “during action observation, children with ASD need greater stimuli variations than TD children to detect their differences in terms of vitality forms” (Di Cesare et al., 2017, p. 8). And, in a study of visual and auditory expression of vitality, Di Cesare et al. (2017) have concluded, “…it may be plausible that visual information is not sufficient for children with ASD to encode vitality forms correctly and that the use of alternative (additional) perceptual information may help vitality form perception” (p8, italics added). As such, Rhythmic Relating consciously supports understanding of vitality through: clarity and contiguity through isolation of copied movements; exaggerated and diminished copying where appropriate; multi and cross modal vitality matching; and the use of acoustic sensory impetus (through rhythm, accents, and acoustic activation contours) to give extra dimensionality and energy to vitality expressions (for further analysis see Daniel et al., 2022).

^14^ https://www.thempra.org.uk/social-pedagogy/key-concepts-in-social-pedagogy/the-common-third/

^15^Of course, if we know our partner well, there will be exceptions to this. There will be many circumstances where touch is playful and appropriate and flowing consent is clear. There will be circumstances where very direct language, like “it’s your turn’ will be helpful and dynamic.

^16^As we are suggesting practical use of rhythmic experiences, and a reliance on a felt sense of a repetitious behavioural pattern, it is important that we address potential beat-perception in people on the autism spectrum. Across simple and complex meter conditions, pre-SMA, basal ganglia, and cerebellar dysfunction in autism appears to be functionally specific, leaving beat-perception largely intact (Rinehart et al., 2006; Qiu et al., 2010; DePape et al., 2012). The anatomically and functionally specific nature of basal ganglia dysfunction in particular (Rinehart et al., 2006; Qiu et al., 2010) may likely allow for a window of beat-based experience and support (for detailed analysis, see Daniel et al., 2022).

^17^No studies have been identified that establish equivalence for individuals with autism. Consequently, we will move forward with the provisional working assumption that the six-second window is suitable for the upper range of a sense of rhythm. In practical terms, it is crucial for the practitioner to maintain their awareness of the ongoing rhythm, making the six-second estimate entirely applicable in this context.

^18^Our concepts here, are closely akin to Feldman’s *matching synchrony* and *sequential synchrony* (Feldman, 2007a,b).

^19^Such “autism motor signatures” can be computationally identified from 2½ years of age (Anzulewicz et al., 2016).

^20^Stern (1999) described motor contours in action as, *vitality contours*. Our concept of *activation contours*, encompasses this movement concept but also integrates the inclusion of compelling contours of sound.

^21^The ASR operates along a pathway leading directly from the cochlea, along cranial (auditory) nerve VIII by way of the lateral leminiscus, to the caudate reticular nucleus. From here, there are descending projections to spinal and limb motor neurons, provoking the “jump” or “blink” effect (Frankland et al., 1997; Osborne, 2009).

^22^It is very likely that these sounds are recognized by innate systems early in auditory pathways (Erlich et al., 2013; re: the Inferior Colliculus, Jorris et al., 2004; Sivaramakrishnan et al., 2004; Osborne, 2009). There is clear evidence of these pathways ascending to emotional systems (Heldt and Falls, 2003), as well as a descending, emotional “feedback” pathway from the amygdala (Marsh et al., 2002).

^23^Autistic individuals have demonstrated prolonged *unmodulated* ASR latency as compared with age-matched typically developing controls (though without controls for learning disability) (Ornitz et al., 1993). Understanding the conditions which *modulate latency* is important for us here as, if we can minimize relative autistic-non-autistic latency difference, we can improve alignment for synchrony. When startle stimuli were presented with *pre-stimulation* and/or *with habituation*, latency differences and auditory hypersensitivities (shown via ASR amplitude) became non-significant (Ornitz et al., 1993) Clearly, predictive information - through context and familiarity - matters. In addition, autistic participants were slower to gain the benefits of habituation, needing a longer period before ASR latency effects were minimized, as compared with age-matched typically developing controls (Ornitz et al., 1993). So, repetition and patience matter too.
